# Central Nervous System Involvement in Adult Acute Lymphoblastic Leukemia: Diagnostic Tools, Prophylaxis, and Therapy

**DOI:** 10.4084/MJHID.2014.075

**Published:** 2014-11-01

**Authors:** Maria Ilaria Del Principe, Luca Maurillo, Francesco Buccisano, Giuseppe Sconocchia, Mariagiovanna Cefalo, Giovanna De Santis, Ambra Di Veroli, Concetta Ditto, Daniela Nasso, Massimiliano Postorino, Marco Refrigeri, Cristina Attrotto, Giovanni Del Poeta, Francesco Lo-Coco, Sergio Amadori, Adriano Venditti

**Affiliations:** 1 Ematologia, Dipartimento di Biomedicina e Prevenzione, Università Tor Vergata, Roma, Italia.; 2 Istituto di Farmacologia Translazionale, Dipartimento di Medicina, CNR, Roma, Italia.; 3 Fondazione S. Lucia

## Abstract

In adult patients with acute lymphoblastic leukemia (ALL), Central Nervous System (CNS) involvement is associated with a very poor prognosis. The diagnostic assessment of this condition relies on the use of neuroradiology, conventional cytology (CC) and flow cytometry (FCM). Among these approaches, which is the gold standard it is still a matter of debate. Neuroradiology and CC have a limited sensitivity with a higher rate of false negative results. FCM demonstrated a superior sensitivity over CC, particularly when low levels of CNS infiltrating cells are present. Although prospective studies of a large series of patients are still awaited, a positive finding by FCM appears to anticipate an adverse outcome even if CC shows no infiltration. Current strategies for adult ALL CNS-directed prophylaxis or therapy involve systemic and intrathecal chemotherapy and radiation therapy. An early and frequent intrathecal injection of cytostatic combined with systemic chemotherapy is the most effective strategy to reduce the frequency of CNS involvement. In patients with CNS overt ALL, at diagnosis or upon relapse, allogeneic hematopoietic stem cell transplantation might be considered. This review discusses risk factors, diagnostic techniques for identification of CNS infiltration and modalities of prophylaxis and therapy to manage it.

## Introduction

Over the last two decades, clinical trials have generated improved response rates in adult patients with acute lymphoblastic leukemia (ALL). Advances in understanding disease biology, adoption of induction and maintenance programs based on risk-adapted strategies, similar to the treatment in children, and better supportive care, have all contributed to those improvements. Overall, adults with ALL have a 60–90% chance of attaining a first complete remission using combination chemotherapy.^1^–^3^ In this context of a better-controlled systemic disease, central nervous system (CNS) involvement has become an even more influential limitation to achievement of long-term cure and a primary cause of mortality.

CNS may be involved at initial diagnosis or relapse. At initial diagnosis, about 5% of adults has CNS involvement^4^,^5^ being, their duration of overall survival (OS) shorter than the one of those without CNS involvement. The incidence of CNS involvement upon relapse is quite variable. Surapaneni et al^6^ reported a CNS relapse rate of 7% whereas, in the French Leucémie Aiguës Lymphoblastique de l’Adulte (LALA) trials, 15% of the patients developed a CNS relapse^7^. Isolated CNS recurrences range from 0% to 11%,^1^,^8^,^9^ while CNS and bone marrow relapses occur in an additional 1–4% of the patients.^10^ Most patients with isolated CNS recurrence subsequently relapse in the bone marrow too. Although these figures might be underestimated, given the possibility that physicians do not systematically investigate CNS involvement at the time of relapse, CSF analysis and CNS prophylaxis should be mandatory in each treatment protocols. Prognosis of adult patients who experience CNS relapse is very poor with a median OS of six months and a projected 5-year OS of zero.^11^

In the present review, we discuss some aspects of this serious complication, such as risk factors, diagnostic tools, prophylaxis, and therapy.

## Risk Factors for Cns Localization

Several risk factors have been associated with the development of ALL CNS involvement. Age seems to be a key factor with a higher incidence in younger adults^12^. Mature B-cell subtype is also associated with an increased risk of CNS localization. A retrospective analysis by Bassan and colleagues^13^ indicates that adult patients with mature B-ALL have an 18% incidence of CNS involvement at presentation compared with an overall incidence of 4.5%. In contrast, Lazarus et al.^4^ reported a higher incidence of CNS involvement at diagnosis in association with the T-cell immunophenotype. The Philadelphia (Ph) chromosome positivity is also considered as a high-risk signature for CNS leukemia.^14^ Patients with CNS involvement at diagnosis are more likely to have lymph node enlargement, mediastinal mass,^4^,^7^ and other extra-medullary localizations.^7^ Finally, lactate dehydrogenase (LDH) level, white blood cell (WBC) count and proliferative index have been identified as additional risk factors rendering patients prone to CNS relapse. Incorporating elevated LDH, serum β2-microgobulin and high leukemia cell proliferation rate in a multivariate analysis, the colleagues from the M.D. Anderson Cancer Center identified discrete categories of adult patients with different chances to develop CNS leukemia.^14^,^15^ Patients with one risk factor had 13% probabilities to develop CNS disease at 1 year, if two or more risk factors were present probabilities increased to >20%. Above all, the presence of leukemic cells in the cerebrospinal fluid (CSF) is considered the most crucial feature of risk. Traditionally, patients are considered at increased risk of CNS relapse if detection of blast cells in CSF is accompanied by a CSF-WBC count exceeding 5 cells/μl. In 1990s, it was proposed that the presence of any number of blast cells in the CSF, regardless of CSF-WBC count, is associated with an increased risk of CNS relapse.^5^,^16^,^17^ Based on this, a specific risk score was generated: CNS1, denoting the absence of identifiable leukemic cells in CSF; CNS2, denoting the presence of blast cells in a CSF sample containing <5 WBC/μl; and CNS3, a CSF sample that contains ≥5WBC/μl together with identifiable blast cells, or the presence of cerebral mass, or cranial nerve palsy together with leukemic cells in the CSF. An increased incidence of CNS relapse has also been observed when a traumatic lumbar puncture is associated with the presence of blast cell in the CSF. The relevance of this CNS risk score has been subject of dispute since several authors did not find significant differences in outcome, for patients categorized as CNS1 versus CNS2.^18^,^19^ In addition, the clinical significance of traumatic lumbar puncture remains unclear and controversial.^20^

## Diagnostic Tools

CNS involvement in ALL remains under-diagnosed; this is confirmed by the autoptic demonstration of CNS infiltration in patients who, at the onset of ALL, were considered as having bone marrow disease only.^21^ Therefore, a correct and timely diagnosis still represents a challenge. Besides the clinical evaluation of neurological signs and symptoms, three independent techniques are used to diagnose CNS disease in ALL patients: CNS neuroradiology, CSF cytology and flow cytometry examination.^22^

### Clinical evaluation

Clinical manifestations may vary, depending on the size of leukemic infiltration, the sites and number of sites involved. Brain localization scan cause headache, alteration of mental status, walking abnormalities, nausea and vomiting, loss of consciousness, seizures, gait or sensory disturbances, papilloedema. Cranial nerves localization may be associated with diplopia, hearing and visual loss, facial numbness, dysphagia. Spinal involvement can determine focal weakness (of legs more often than arms), paresthesias, back pain, radicular pain, bladder, and bowel dysfunction. The correct interpretation of clinical presentation is often challenging. In fact, neurological symptoms and signs may be subtle, and sometimes attributed to other causes, directly or indirectly related to ALL, such as hyperleukocytosis, metabolic encephalopathy, treatment-related neuropathy, opportunistic infections. In some patients, CNS involvement develops completely asymptomatic and therefore detected by routine lumbar puncture.

### Neuroradiology

A variety of neuroradiographic methods are available to evaluate patients with suspected CNS involvement, including cranial computed tomography (C-CT), gadolinium-enhanced brain and spine magnetic resonance imaging (MRI). C-CT is abnormal in about 25% of patients with carcinomatous meningitis.^22^–^24^ However, the detection power of this technique decreases when it comes to the evaluation of patients with suspected leukemic meningitis, so that positive findings are significantly less than the 25% achievable in solid tumors meningitis.^25^ MRI with gadolinium enhancement has a superior sensitivity than cranial C-CT^24^ and accordingly, it is the radiologic first choice to explore CNS localization of ALL. Since ALL can potentially infiltrates any area of neuraxis, T1-weighted sequences, with and without contrast, combined with fat suppression T2-weighted sequences, represent the standard techniques to scan the entire CNS, in patients for whom localizations are suspected. Indicative of CNS disease are MRI enhancement and/or enlargement of cranial nerves, nodular or linear leptomeningeal enhancement extending into sulci or basal cisterns, and intradural-enhancing nodules, especially those located at the cauda equine. Finally, MRI allows identifying abnormalities, such as leukoencephalopathy, brain atrophy, old hemorrhages or old infarcts, due to treatment but not to disease. Despite its superiority over C-CT, even MRI has some pitfalls. One study found that MRI was capable of detecting 100% of case of neoplastic meningitis due to solid tumor but only 44% of those due to B-cell ALL^26^. It has been estimated that the potential false-negative rate of MRI is as high as 60–65% and the false-positive one about 10%. These data limit the use of MRI as a stand-alone diagnostic tool, and a normal MRI imaging does not provide certainty about the absence of occult CNS disease in the course of ALL.

### CSF examination

CSF examination is the most useful laboratory test in the diagnosis of ALL CNS involvement. Abnormalities include increased opening pressure (>200 mm of H_2_0), elevated protein (>50 mg/dl) and decreased glucose (<60 mg/dl) CSF concentration and increased WBC count (>5/mm^3^), which is not diagnostic but only suggestive of CNS involvement. In infectious diseases, like bacterial and viral meningitis, there may be a marked elevation of WBC count. Besides, some authors observed no significant difference in total protein, glucose and WBC count between patients with CNS localization and patients without.^27^,^28^

The presence of leukemic cells in the CSF is diagnostic for CNS involvement and, if the lumbar puncture is clinically and technically feasible, CSF examination must be performed. CNS leukemia is defined as unequivocal morphologic evidence of leukemic blast in the CSF and/or mononuclear cell count ≥5/μl. Morphologic examination is performed on cytospin preparation stained with May- Grunwald-Giemsa. Conventional cytology (CC) is estimated to have a >95% specificity for CNS involvement. However, it has a relatively low sensitivity (<50%) and consequently is often falsely negative. Low sensitivity of CC is due to paucity of cells in CSF and morphological similarities that can make it difficult to distinguish benign from malignant cells. In the largest postmortem analysis of patients with neoplastic meningitis, Glass et al.^29^ showed that 41% had leukemic meningitis on autopsy but a negative pre-mortem CC. They also demonstrated that, in patients with a focal leptomeningeal disease, the occurrence of cytological false negatives was >50%, emphasizing the frequent co-occurrence of CNS disease and negative CC. In patients with suspected CNS involvement, because of the low detection rate, lumbar punctures are often repeated up to three times. However, even after repeated CSF sampling, false negative cytology reportedly occurs in 10% to 20% of patients with leptomeningeal disease. In a series including lymphomatous and leukemic meningitis Kaplan et al.^30^ found the frequent dissociation between CSF cell count and malignant cytology (29% of cytological positive CSF had concurrent CSF count <4/μl).

Flow cytometric(FCM) immunophenotyping is a valuable tool for the diagnosis and staging of haematological disorders involving lymph nodes, blood, and bone marrow. Clinical flow cytometry assays have been implemented to reliably detect phenotypically abnormal cells representing 0,01% of events (1 cell in 10^4^) and is a useful tool for monitoring minimal residual disease in acute leukemia^31^. Although powerful and extremely sensitive, FCM assay relies on rigorous technical requirements: CSF samples of sufficient volume must be obtained via lumbar puncture. After sampling, CSF should be processed within 1 hour to avoid cell deterioration. In this view, some authors recommend the use of fixative (TransFix/ethylenediaminetetraacetic acid EDTA; Immunostep SL Salamanca, Spain).^32^ The samples should be collected in tubes with no anticoagulant and transferred to the laboratory as quick as possible. To obtain the maximum number of cells for analysis, CSF should be concentrated by low-speed centrifugation^33^. One subject of controversy pertains the threshold defining FCM positivity. Di Noto et al.^34^ use a threshold of at least 30 events; in a less restrictive approach, Qujiano et colleagues^32^ considered a minimum of ten events, shaping a cluster, as a proof of CNS infiltration. Subira et al.^35^ suggest that at least 9 B-cell or 12 T-cell events are required to reach a confidence level of 95%, thus indicating the presence of CNS disease. These results are in agreement with those of Craig et coworkers,^36^ in the experience of whom, at least 13 clustered events displaying identical features are required to identify a specific cell population. In general, the presence of fewer than 5 clustered events is not regarded as related to the presence of a specific population. A qualitative approach might be an alternative to the quantitative one. Rather than defining a numerical threshold, it might be important to take into account how tightly the cells are clustered and whether their characteristics profile a particular disease entity.^31^ The use of a cocktail of 6–9 monoclonal antibodies represents a further strategy to increase FCM sensitivity and enhance qualitative information achievement.^37^ Based on the above-mentioned considerations, FCM is considered to be more sensitive than CC for the detection of malignant hematologic cells in CSF.

A number of studies published in recent years, dealing with detection of CNS disease in ALL or newly diagnosed aggressive non-Hodgkin’s Lymphomas, demonstrated the superior sensitivity of FCM over standard cytology.^27^,^32^,^34^,^38^ In a retrospective analysis of CSF samples collected from 219 patients with leukemia/lymphoma, FCM discovered CNS infiltration in 44 patients, of these only 19 were positive by CC. Patients with a positive finding by CC had a higher incidence of neurological signs and symptoms and CSF pleocytosis.^28^ FCM characterizes for the ability to reveal hematologic disease in CSF specimen even when cellularity is very low.^36^,^39^ This peculiarity has been confirmed in pediatric ALL patients where FCM was able greatly to improve the recognition of occult CSF involvement.^40^ Mitri et al.^41^ applied FCM to 267 CSF samples obtained from 80 adult ALL patients and fund that FCM had 100% sensitivity and specificity in detecting lymphoblasts. The authors concluded that additional information is needed to determine the clinical significance of a single FCM positivity. In fact, in the absence of morphologically evident blasts on CC, it is still a matter of debate whether or not the FCM positivity affects clinical outcome in ALL. Although Mitri et al. analyzed a consistent number of samples, one would argue that they provided no information whether or not their patients belonged to a consecutive series. In addition, they analyzed CSF samples in a 4-color assay which, on a technical ground, might not be appropriate to detect rare events. These observations may explain why Mitri et al.^41^ found a positive CNS sample with FCM only in 1.5% of newly diagnosed cases whereas we^42^ and others^43^ have found in 24% and 28%, respectively (Table 1).

**Figure 1:**
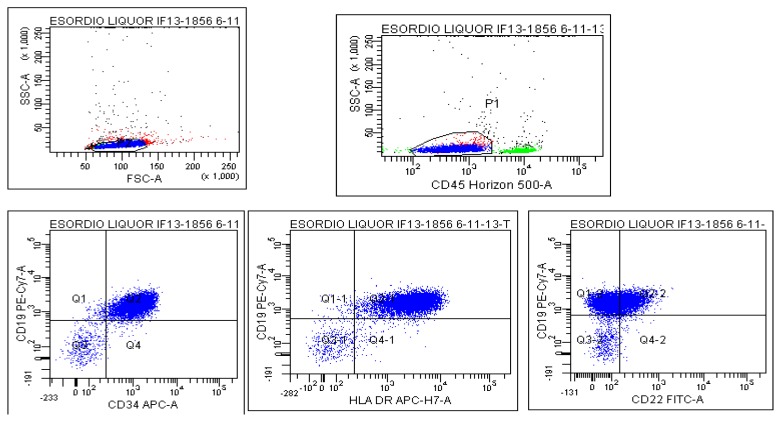
Flow cytometry detection of blast infiltration of cerebrospinal fluid in a patient with B Acute Lymphoblastic Leukemia. The leukemic population is depicted in blue which denotes cluster of cells expressing CD19, CD34, CD22 and HLA-DR.

In patients affected with high-risk non-Hodgkin lymphomas and Burkitt’s lymphomas, a single FCM positivity of CSF was associated with a significantly higher risk of CNS relapse and a worse prognosis.^44^,^45^ One hundred and 68 CSF samples taken from 31 patients with ALL were analyzed by FCM and conventional cytology. In all samples findings were concordant but in 10, results of which were discrepant. However, all patients with negative FCM results remained free from CNS disease.^35^ In a population of 38 adults with ALL or lymphoblastic lymphoma, we confirmed that FCM was more sensitive than CC in recognizing CSF localization (Figure 1). In our study, CC failed to identify the presence of neoplastic cells in 9/14 (64%) FCM positive patients, and 3 (33%) of these 9 developed an overt CNS disease. None of the FCM negative patients experienced such a progression. Furthermore, the median overall survival of patients with a single FCM positivity was intermediate between patients double positive and negative.^42^ Consistently, the molecular CSF detection of a leukemic signature in pediatric patients correlated with a shorter 4-year event-free survival compared with those without such a signature.^45^ In a multicentric prospective study of children with ALL, Martinez-Laperche et al.^43^ demonstrated that identification by FCM of subclinical leukemic infiltration of CSF during maintenance correlated with a significantly shorter duration of 3-years relapse-free and overall survival. However, despite the efficient sensitivity of FCM, complementary diagnostic approaches might be required to solve cases such as those with neurological symptoms but with no radiological or cytometric evidence of CNS disease. In this regard, it has been demonstrated that quantification of soluble CD19 represents a surrogate biomarker for occult CNS lymphoma,^47^ paving the way to its assessment even in B-ALL.

### Prophylaxis of CNS localization

Due to the limited penetration of cytostatic drugs across the blood-brain barrier into the CSF and brain parenchyma, CNS represents a sanctuary site. Blood-brain barrier (BBB) is a highly specialized network where interactions between astrocytes and vascular endothelium counteract delivery of many chemotherapeutic agents. The insufficient CNS accumulation of the drugs conventionally used to treat ALL explain why, in absence of adequate prophylaxis, recurrence at this site is observed in approximately 30% of adult patients.^48^ Standard CNS prophylaxis in ALL relies on the combined use of systemic and intrathecal (IT) chemotherapy or radiation therapy.

### Systemic chemotherapy

The prophylactic role of systemic chemotherapy is strictly dependent on factors such as the ability of the drugs to cross the BBB and to distribute uniformly within the parenchyma, and their active extrusion from CNS. The ability of high-dose cytarabine (ARAC) and metotrexate (MTX) to penetrate the BBB makes them suited agents for CNS prophylaxis in ALL.^48^,^49^ MTX is the most widely used hydrophilic chemotherapeutic agent, but high doses must be administered to achieve therapeutic drug concentration in CNS. The bolus intravenous injection increases brain delivery of MTX compared with the slow intravenous infusion. With the use of calcium folate based rescue, very high systemic doses of MTX (5–8 g/m^2^) can be administered safely, and therapeutic levels can be achieved despite its limited capability of CSF penetration. High-dose ARAC has also been successfully used for CNS prophylaxis. Since the ARAC half-life in CSF is 8-fold greater than in plasma, prolonged cytotoxic concentrations can be achieved with doses of 3 g/m^2^ given every 12 hours. Cortes et al.^50^ demonstrated the efficacy of the combination of high-dose of both MTX and ARAC with the adjunct of IT ARAC, to prevent CNS recurrence in adult patients with ALL. Although MTX and ARAC were identified as the most effective drugs for systemic CNS prophylaxis, no agreement has been reached on the optimal doses and number of cycles at which they should be delivered. In the Cortes’ study,^50^ MTX dose might be too low for an effective CNS penetration whereas that of ARAC too high in terms of toxicity. Current approaches favor the use of higher MTX (2.5–3 g/m^2^) and lower ARAC (2 g/m^2^) doses. Steroids have also been extensively used. Dexamethasone concentration can reach higher CSF levels and has a longer half-life than prednisone.^51^,^52^ Annino et al.^53^ reported that the addition of high-dose of dexamethasone to systemic treatment reduces the rate of CNS recurrence to 2%. Systemic etoposide^54^ and 6-mercaptopurine^55^ can also reach adequate concentrations in CSF, as well as systemic administration of L-asparaginase can result in prolonged CSF depletion of L-asparagine.^56^ In childhood ALL, delivery of Erwinia-derived asparaginase was associated with CNS relapse at a nearly six times rate than patients treated with Escherichia coli-derived asparaginase.^57^ The experience with the use of systemic chemotherapy indicates that, when given alone, it is not sufficient for CNS prophylaxis. This is mainly due to the difficulties to maintain persistent drugs concentration while in presence of remarkable side effects (neurotoxicity, mucositis, diarrhea, fever, liver dysfunction) associated with administration of high-dose MTX and/or ARAC.

### Intrathecal chemotherapy (IT)

IT chemotherapy is the preferred method for CNS prophylaxis. Commonly used IT therapies include injection of MTX, ARAC, and liposomal ARAC. MTX has always been considered superior to ARAC because it persists longer in the CSF and penetrates more deeply into meninges and CNS parenchyma.^58^ MTX dose can be variable with some authors suggesting 12.5 mg,^3^ others 15 mg.^7^,^59^ It can be given either alone or in conjunction with ARAC and hydrocortisone or methylprednisolone. It was thought that the combination of MTX with ARAC may have additive or synergistic effects, with the role of corticosteroids being the one to attenuate arachnoiditis associated with MTX/ARAC administration. ARAC is the second most widely used agent for IT prophylaxis. It is usually injected at doses of 30 mg/m^2^, which achieves peak concentrations of up to 1 mM.^60^ After IT injection of ARAC, conversion to the inactive metabolite uracil arabinoside is negligible, because of the significantly low cytidine deaminase activity in the brain and CSF; this enhances a longer half-life of ARAC in CSF than in plasma. Usually, IT chemotherapy is initiated early during induction therapy and continued throughout the maintenance. The number of IT injections is variable. In the LALA trials, CNS prophylaxis consisted of 6–8 IT injections of ARAC and MTX, plus or minus methylprednisolone (40mg), in patients receiving only chemotherapy, and 5 IT injections in those also transplanted.^3^,^7^ In the HypeCVAD program, 16 IT treatments were planned^2^. More recently, IT liposomal ARAC has been used for the prophylaxis of CNS malignant involvement. ARAC is encapsulated in a multivescicular liposome preparation named DepoFoam, and the product is known as DTC-101 or DepoCyt^61^. This encapsulation modifies the pharmacokinetics of the free ARAC released in CSF in a way that the cytotoxic concentration of the drug is maintained for as long as 14 days. A phase II randomized trial of radiation–free CNS prophylaxis, comparing IT triple therapy (methotrexate 12.5 mg, cytarabine 50mg, prednisone 40mg) with liposomal ARAC (50mg), showed that liposomal ARAC was feasible and at least as effective as other regimens.^62^

In the adult ALL German Multicenter Study Group prospective trial, liposomal ARAC confirmed its safety and effectiveness even in the subgroup of older (>55 years) Ph-negative patients. Analysis of efficacy indicated that CR was increased, and mortality decreased in the arm receiving IT liposomal ARAC likely due to a less pronounced bone marrow toxicity^63^.

### Radiation Therapy

Although cranial (CI) and/or cranio-spinal irradiation (CSI) is the oldest approach for CNS prophylaxis in pediatric patients with ALL,^64^,^65^ few studies have systematically explored its prophylactic role in adults. In the prospective trial of Southeastern Cancer Study Group, random assignment to CNS prophylaxis, including CI, or not resulted in a significant prolongation of CNS relapse-free interval for patients receiving CNS prophylaxis.^66^ Sanders et al.^67^ reported the effectiveness of CSI in preventing CNS recurrence in adult patients who achieved complete remission. Although CI/CSI can be an effective form of CNS-directed therapy it is often associated with late adverse effects, such as endocrinopathy, neurocognitive dysfunction, and neurotoxicity. These side effects are fewer and less pronounced in adults than in children, although patients aged >60 years appear to be more susceptible than younger to cognitive impairment. It remains not clarified what dosage of CI/CSI and what prophylaxis strategy is the best. Twenty-four grays are the standard prophylactic dose for CI in combination with IT-MTX. Others found that a dose of 18 grays is equally effective.^68^ There have also been attempts to omit CI in clinical trials of adult patients. Kantarjian et al.^2^ reported that systemic MTX and ARAC plus IT-MTX reduced the rate of CNS recurrence to 4%, with no need of CI/CSI. Furthermore, in a study recruiting a series of 467 adult patients who received IT and high-dose of systemic therapy, but not CI, the frequency of CNS recurrence was similar to that observed in protocols including prophylactic CI^59^ The phase 2 study 19802, from Cancer and Leukemia Group B (CALGB), demonstrated that the combination of high-dose systemic and IT MTX can substitute for CI. In fact, isolated CNS relapses occurred in 6% of the patients, a rate that is comparable to the one of four prior CALGB studies including CI.^69^

## Therapy of CNS Localization

CNS prophylaxis in adults with ALL determines a reduction of CNS localization by 20–30%. Nevertheless, about 10% of subjects who are diagnosed with ALL eventually develop overt CNS disease. Although such a circumstance connotes a very adverse prognosis, the available therapeutic options are as the same as those used for CNS prophylaxis. As a consequence, strategies such as more frequent IT treatments and intensification of systemic chemotherapy have been adopted. In the LALA trials,^3^,^7^ patients with CNS involvement at diagnosis were treated with 18 double (ARAC plus MTX) or triple (ARAC, MTX and methylprednisolone) IT injections associated with a pre-transplant CI of 15–20 grays. In the absence of HSCT, patients received a 24 grays CI. When compared with MTX or ARAC administered twice a week, liposomal ARAC has a similar safety profile and same or even better effectiveness in the treatment of lymphomatous meningitis.^70^ Side effects commonly associated with liposomal ARAC include headache, arachnoiditis, and confusion; to mitigate the occurrence of arachnoiditis, liposomal ARAC should be given in conjunction with dexamethasone.^71^ Because of the occurrence of severe neurotoxicity, an additional precaution, and strict surveillance should be adopted when IT liposomal ARAC and BBB penetrating systemic agents are administered simultaneously or in close sequence.^72^ In a phase 2 European trial, 19 patients with isolated or bone marrow associated CNS relapse were treated with liposomal ARAC and systemic chemotherapy. Liposomal ARAC was administered at dosage of 50 mg on day 1 and continued with an administration every 14 days for a maximum of five additional injections. Early complete cytological remission of CSF was observed in 74% of the patients.^73^ It has been observed that patients with CNS overt leukemia at diagnosis, by intensifying the therapy, have a similar outcome than those who did not present with this condition.^7^ In the international MRC UKALLXII/ECOG 2993 trial, Lazarus and coworkers^4^ observed CNS involvement in 77 of 1508 (5%) adult patients with ALL. In addition to treatment by protocol, these 77 patients received IT or intra-ventricular MTX (12.5 mg three times/week) followed or not, at physicians’ discretion, by CI. CI or CSI were administered at dosage of 24 and 12 grays, respectively. After induction and intensification, all patients were recruited to receive either consolidation/maintenance or allogeneic hematopoietic stem cell transplantation. Complete remission rate in patients with or without CNS disease was comparable (90%) whereas 5-year overall survival rate was 29% and 38%, respectively (p=.03). The authors concluded that both allogeneic hematopoietic stem cell transplantation and chemotherapy intensification are valid options to improve outcome of patients with active CNS disease at diagnosis. Finally, it should be pointed out that the therapeutic role of CI/CSI is not clearly defined as the prophylactic one. It is very marginal when the CNS involvement occurs as a relapse in patients who have already been irradiated. In this situation, it should be delayed until completion of systemic chemotherapy.

### Ph-chromosome positive ALL

Treatment of Ph-positive ALL has been subjected to substantial changes since the introduction of BCR-ABL tyrosine kinase inhibitors (TKI). Exploring to what extent the use of TKI might prevent CNS localization of ALL has been a major point of interest. Imatinib is the first generation TKI approved for the treatment of patients with Ph-positive ALL and, despite its use, up to 20% of treated patients develops CNS relapse.^74^ In many cases, these relapses occur in patients with morphologic complete remission^74^ and have been attributed to the insufficient penetration of imatinib into the CSF.^75^ Dasatinib, a second-generation TKI of SRC-kinase and BCR-ABL, has shown significant activity in adults with imatinib-resistant or -intolerant Ph-positive ALL.^76^ BBB penetration of dasatinib was observed in pre-clinical mouse models of intracranial Ph-positive leukemia and in pharmacokinetic studies of a series of 22 patients with Ph-positive ALL or chronic myeloid leukemia.^77^ Detectable levels of dasatinib were found in only in 6 (2 adults and 4 children) of these 22 patients, thus its reported clinical activity in CNS localization of Ph-positive ALL is anecdotic and still awaits for a formal demonstration. Similar to dasatinib, nilotinib, is a second-generation TKI which in preliminary studies has demonstrated activity in treating CNS localization of Ph-positive leukemia.^78^ Hypothetic reasons for nilotinib activity rely on its pharmacokinetic profile. In fact, nilotinib has a high protein-binding affinity, which contrasts with the low protein concentration in CSF; this condition is supposed to translate into a relatively higher amount of free and therefore active nilotinib in CSF than in blood.^78^ Finally, aggregation studies have indicated that imatinib and dasatinib do interfere with platelets function whereas nilotinib does not.^79^ This might have practical implications in thrombocytopenic patients. Among ten adults with Ph-positive ALL receiving imatinib, Patel et al.^80^ described 3 instances of subdural hematomas occurring after IT injection of chemotherapeutic agents. Given the apparent lack of effect of nilotinib on platelet aggregation, the authors suggest that this TKI should be considered for combination therapies including systemic and IT delivery of cytotoxic drugs.

### Chimeric antigen receptor (CAR)

Engineered CAR-T cells targeting CD19 or CD20 antigens are emerging as powerful therapies in hematologic B-malignancies, and CAR-T cells were found in CSF of several patients recruited to dedicated trials.^81^,^82^ CAR-T cells presence in CSF might be due to the enhanced cell trafficking through BBB promoted by IL6 release following CART infusion.^83^ Alternatively, authors have claimed that some cross-reactivity or undetectable expression of CD19 in the brain might trig CAR-T cells migration to CSF^81^. Whatever the reason is underlying the presence of CAR-T cells into CSF, an open question remains whether these might have a role in eradicating CNS disease. Lee et al.^84^ reported that in 3 of eight patients treated for a diagnosis of refractory B-malignancies, CAR-T cells were detected in CSF. Of these 3, one with a stage CNS2 at the time of trial enrollment cleared all CSF blasts as demonstrated by flow-cytometry. Very recently, it has been shown in an ALL pediatric population that CAR-T cells were detectable in CSF, and that 2, whose CSF contained blast cells at the time of CAR-T infusion, became subsequently free of CNS.^85^

## Conclusions

In ALL, effective CNS clearance requires adequate systemic and/or IT prophylaxis and therapy. The devastating effects of CNS relapse and the subsequent intensive CNS-directed therapy both require that the patients are properly stratified in order to avoid over and undertreatments. Owing to its superiority over CC in detecting even low levels of infiltrating cells, FCM may well serve the purpose of risk-stratification and should therefore become a routine tool for diagnostic assessment of ALL. Further and large studies are needed to standardize the procedures and permit an optimal clinical application of this technique.

## Figures and Tables

**Table 1:** Comparison of FCM and CC for detection of leukemic cells in CSF of ALL patients

STUDY	N^∘^	Positive FCM	Positive CC
*Sayed (2009)	45	21(46%)	10(22%)
**Martinez-Lapalerche (2013)	108	30(28%)	3(3%)
^ Mitri (2014)	80+15	1/66(1.5%) + 5/15(33%)	1/80(1.2 %) + 5/15(33%)
^^ Del Principe (2014)	38	14(24%)	5(13%)

FCM indicates flow cytometry; CC, conventional cytology; CSF, cerebral fluid spinal; ALL, acute

* Pediatric Patients: 12 pts with neurological abnormalities, 33 pts without symptoms, whom, 24 at first presentation and 9 at relapse.

** Pediatric Patients at diagnosis without neurological abnormalities

^ Adult: 80 Patients at diagnosis without neurological abnormalities + 15 Patients at relapse

^^ Adult patients at diagnosis without neurological abnormalities
